# Safety of unconventional antibody-drug conjugate L-DOS47 in a phase I/II monotherapy study targeting advanced NSCLC

**DOI:** 10.3389/fonc.2025.1544967

**Published:** 2025-08-11

**Authors:** Rodryg Ramlau, Dariusz M. Kowalski, Aleksandra Szczęsna, Cezary Szczylik, Young Ou, Martin Köbel, Gabrielle M. Siegers, Kim J. Gaspar, Brenda Lee, Kazimierz Roszkowski-Sliz

**Affiliations:** ^1^ Center of Pulmonary and Thoracic Surgery, Med-Polonia Sp. z o.o., Poznań, Poland; ^2^ Department of Lung Cancer and Chest Tumors, Maria Sklodowska-Curie National Research Institute of Oncology, Warsaw, Poland; ^3^ Department of Lung Diseases, Oncology Division, Mazowieckie Centrum Leczenia Chorób Płuc i Gruźlicy, Otwock, Poland; ^4^ Department of Oncology, Europejskie Centrum Zdrowia Otwock, Otwock, Poland; ^5^ Department of Pathology and Laboratory Medicine, University of Calgary, Calgary, AB, Canada; ^6^ Helix BioPharma Corp, Toronto, ON, Canada; ^7^ Third Department of Lung Diseases, National Tuberculosis and Lung Diseases Research Institute, Warsaw, Poland

**Keywords:** tumor microenvironment, ADC, nanobody-drug conjugate, CEACAM6, CEACAM-6, acidosis

## Abstract

**Introduction:**

Tumor acidity is emerging as a hallmark of cancer and carcinoembryonic antigen-related cell adhesion molecule 6 (CEACAM6) expression is frequently increased in acidic tumors. L-DOS47, a novel antibody-drug conjugate (ADC), consists of jack bean urease cross-linked to anti-CEACAM6 nanobodies. L-DOS47 is thus both targeted to CEACAM6-expressing tumors and designed to improve tumor control by neutralizing the acidic tumor microenvironment (TME) through ammonia and bicarbonate production from local urea.

**Methods:**

This open-label, non-randomized study evaluated safety and tolerability of L-DOS47 in Stage IIIB/IV non-small cell lung cancer (NSCLC) patients. The Phase I 3 + 3 dose escalation aimed to determine the maximum tolerated dose (MTD) of L-DOS47 administered once/week over 14 days followed by seven days rest. Phase II explored twice-weekly dosing.

**Results:**

55/90 patients enrolled in Phase I received L-DOS47 up to 13.55 μg/kg. Although one dose-limiting toxicity (DLT) occurred in a patient at 5.76 μg/kg, MTD was not reached. Common treatment-emergent adverse events (TEAEs), reported by 38% of patients, were respiratory/thoracic/mediastinal disorders including dyspnea. No complete (CR) or partial responses (PR) were observed in Phase I or Phase II, despite the latter’s intensified dosing regimen; however, Phase I *post-hoc* exploratory analyses found that progression-free survival (PFS) was significantly extended at doses ≥5.76 μg/kg (P=0.0203). Anti-L-DOS47 antibody (ADA) titers were not associated with AE or shorter PFS. Immunohistochemistry (IHC) screening of an unrelated cohort revealed high CEACAM6 expression in 45.2% NSCLC cases.

**Conclusions:**

L-DOS47 monotherapy was well tolerated at doses up to 13.55 μg/kg. No CRs or PRs were observed but extended PFS was associated with higher doses. Screening for CEACAM6 expression may select patients who are more likely to derive benefit from L-DOS47.

**Clinical Trial Registration:**

https://www.clinicaltrialsregister.eu/ctr-search/search; EudraCT Identifier: 2010-020729-42 (May 6, 2010).

## Highlights

- A novel antibody-drug conjugate that targets tumors expressing CEACAM6, L-DOS47 was designed to combat tumor acidity, for which no approved therapies currently exist.
- L-DOS47 monotherapy was safe, well tolerated, and extended progression-free survival at higher dose levels in heavily pre-treated non-small cell lung cancer patients.
- Screening patient tumors for CEACAM6 expression may help identify patients more likely to benefit from L-DOS47 therapy.

## 1 Introduction

Despite therapeutic advances, long-term prognosis for NSCLC patients remains poor. Although significant gains have been made using immune checkpoint inhibitors, traditional chemotherapy often remains necessary, particularly with advanced disease. Growing evidence supporting the importance of acidosis in cancer development and progression suggests that its reversal in the tumor microenvironment (TME) might improve outcomes (1, 2).

Solid tumors generate a hypoxic environment and cancer cells maintain a glycolytic metabolism even in the presence of oxygen (Warburg effect), causing acid accumulation in the TME (3) that promotes genetic instability, abnormal lipid metabolism, extracellular matrix remodeling, angiogenesis, and metastasis (4–9). Acidity also contributes to local immunosuppression and reduces the efficacy of weakly basic chemotherapy drugs (10–13).

Responses to chemotherapy and immune checkpoint inhibitors have shown improvement in preclinical studies that manipulated TME acidity either directly through oral bicarbonate administration (14), indirectly by inhibiting metabolic proteins that contribute to extracellular proton transport such as Na^+^/H^+^ exchangers, carbonic anhydrases, vacuolar-type H^+^ ATPases, and monocarboxylate transporters, or by targeting increased tumor glycolytic activity (15–18). Despite encouraging preclinical results, however, these agents have not progressed to approval, hence a more targeted approach is needed (4, 18–22).

Antibody-drug conjugates (ADC) have also emerged as potential new targeted cancer therapies, offering antibody-mediated drug delivery to tumor cells that express a specific marker. This is followed typically by internalization of the ADC and release of the cytotoxic payload, which is generally a small-molecule drug. While a number of ADC are in the early stages of clinical development for NSCLC therapy and a few are in late-stage development or pending regulatory approval, only two ADC are currently approved for NSCLC: trastuzumab deruxtecan (T-DXd) for unresectable HER2-positive solid tumors including NSCLC and telisotuzumab vedotin (EMRELIS™), which recently received accelerated FDA approval for NSCLC with high C-met overexpression (23, 24).

L-DOS47 is a novel immunoconjugate consisting of jack bean urease conjugated to approximately 10 copies of a camelid VHH (variable domain of heavy chain of heavy chain-only antibodies) monoclonal antibody, also known as a single-chain antibody or a nanobody, that binds to CEACAM6 with high affinity (25, 26). A glycosylphosphatidylinositol-anchored cell adhesion molecule, CEACAM6 overexpression is associated with altered adhesion and invasion in many cancers, including NSCLC and pancreatic adenocarcinoma (27–29). Although L-DOS47’s mechanism of action is not intended to trigger downstream effects via CEACAM6, recent studies have suggested that CEACAM6 is a potential therapeutic target for oncology drugs and may act as a biomarker for tumor progression in cancers, including lung adenocarcinomas (27, 30, 31). Although CEACAM6 is expressed in both cancerous and normal cells, in immunohistochemical studies the pentameric form of the anti-CEACAM6 nanobody component of L-DOS47 bound strongly to lung adenocarcinoma tissues but not to normal lung tissues, either around lung carcinomas or normal non-cancerous lung tissue (32). Similarly, the NEO-201 monoclonal antibody has been shown to bind to tumor-associated variants of CEACAM5 and CEACAM6 in cancerous but not normal tissue (33, 34). This supported the use of this anti-CEACAM6 antibody as a targeting component in L-DOS47 development for treatment of CEACAM6-expressing cancers with reduced risk of off-target effects.

Unlike conventional ADC, intact L-DOS47 binds to CEACAM6 and remains on the cell surface where the urease converts local urea to ammonia and bicarbonate, raising the TME pH and reversing acidosis (35). In preclinical studies, the urease component of L-DOS47 proved cytotoxic to A549 human lung adenocarcinoma cells *in vitro* and intratumoral administration inhibited tumor growth *in vivo* (36). Furthermore, CEST-MRI studies have shown that L-DOS47 can increase the pH of acidic tumors and also effectively control pancreatic adenocarcinoma growth *in vivo*, especially in combination with an anti-PD1 inhibitor (25, 35). In a Phase I clinical trial in patients with recurring or metastatic NSCLC, L-DOS47 in combination with pemetrexed and carboplatin was well tolerated with encouraging anti-tumor activity (37).

Here, we report the first-in-human clinical study of L-DOS47 monotherapy. The Phase I dose escalation was intended to determine the MTD of weekly L-DOS47, followed by an exploratory Phase II that employed twice-weekly dosing. L-DOS47 pharmacokinetics and pharmacodynamics were also evaluated. To assess the prevalence of CEACAM6 expression in tumor tissues, immunohistochemistry screening was performed *post hoc* on a tumor microarray (TMA) comprising an unrelated cohort of NSCLC patients.

## 2 Materials and methods

### 2.1 Patient selection

Eligible patients were adults (≥18 years old) with histologically confirmed chemo-naïve or refractory Stage IIIB or IV non-squamous NSCLC with an Eastern Cooperative Oncology Group (ECOG) performance status 0–2, life expectancy of 3 months or more, and adequate organ function (absolute neutrophil count ≥ 1.5 x 10^9^/L, platelet count ≥ 100 x 10^9^/L, hemoglobin ≥ 9 g/dL, creatinine clearance ≥ 60 mL/min or serum creatinine 1.5x upper limit of normal, serum aspartate aminotransferase and serum alanine aminotransferase ≤ 3x upper limit of normal or ≤ 5x upper limit of normal if liver abnormalities are due to underlying malignancy, total bilirubin ≤ 1.5x upper limit of normal). The main exclusion criteria included known history of central nervous system metastatic disease and receiving chemotherapy in the 30 days preceding study start or radiotherapy (except symptomatic treatment of bone metastases), targeted therapy, hormonal therapy or immunotherapy, or major surgery or other study drugs within 4 weeks of study treatment initiation. Other exclusions included history of other malignancies (except non-melanoma skin cancer), sustained QT corrected (QTc) with Frederica’s correction > 450 ms at screening or history of additional risk factors for Torsade de pointes, and taking steroids (other than inhalers or topical steroids) or other medications to suppress the immune system.

### 2.2 Study design

This multicenter, open-label, non-randomized Phase I/II study (EudraCT #2010-020729-42) was conducted at four sites in Poland. The primary objective of Phase I was to define the MTD of L-DOS47 monotherapy administered intravenously once weekly at doses up to 13.55 μg/kg. The primary objective of Phase II was to assess preliminary efficacy of the selected dose and regimen. Secondary objectives of both phases were to evaluate the pharmacokinetics and immunogenicity of L-DOS47 and to evaluate safety and tolerability of multiple doses of L-DOS47.

Tumor response was assessed as per response evaluation criteria in solid tumors (RECIST) criteria v1.1. All radiological assessments to assess eligibility were conducted within three weeks of treatment initiation. Radiologic examinations were performed every other treatment cycle until disease progression.

### 2.3 Study treatment

For Phase I, patients were recruited into 16 cohorts from 0.12–13.55 µg/kg using the standard 3 + 3 dose escalation design (**Supplementary Figure S1A**). L-DOS47 was administered as a weekly 30-minute IV infusion over 14 days followed by seven days rest, repeated for a total of four cycles. Patients received L-DOS47 thereafter if there was sustained clinical benefit and treatment was well tolerated according to the Investigator. To monitor possible infusion reactions and/or allergic reactions, the first cohort was treated eight days before subsequent patients were dosed.

Safety data was reviewed after patients received one cycle of L-DOS47 before dose escalation for successive cohorts. Dose limiting toxicities (DLT) were defined as any National Cancer Institute (NCI) Common Terminology Criteria Adverse Events (CTCAE) v4.0 ≥ Grade 3 non-hematologic and any ≥ Grade 4 hematologic AE that was possibly, probably, or definitely related to L-DOS47 and occurred ≤ 3 weeks after commencing L DOS47 treatment, in the opinion of the Investigator. If two or more DLTs were observed at any dose level ≤ 3 weeks after start of treatment, dosing was stopped and the MTD would be defined as the preceding dose level. If only three patients had been treated at the preceding dose level, that dose would be identified as the tentative MTD. A tentative MTD became final when a total of six patients were treated with fewer than two DLTs observed. Since MTD was not reached with weekly dosing at 13.55 μg/kg L-DOS47, this dose level was chosen as the recommended Phase II dose (RP2D) but with twice weekly administration due to L-DOS47’s apparent short half-life (**Supplementary Figure S1B**).

Treatment was discontinued due to disease progression, allergic reaction, grade 3 or 4 toxicities that required treatment discontinuation, intolerable AE, pregnancy, or patient or investigator decision. AE were graded as per CTCAE version 4.0.

### 2.4 Study endpoints and assessments

The primary endpoint of Phase I was incidence and severity of drug-related AE as per DLT criteria. The Phase II primary endpoint was overall response rate (ORR) (RECIST v.1.1). Secondary safety endpoints for both phases included L-DOS47-related toxicity over two hours post-infusion, incidence and severity of AEs and SAEs, changes from baseline in physical examination and additional safety parameters such as clinical laboratory assessments, vital signs, weight, oxygen requirement and 12-lead electrocardiogram; physical examination; and evaluation of ADA and pharmacokinetics (PK). Additional secondary endpoints for Phase II also included time to disease progression/PFS, and overall survival. PFS was computed as the elapsed time between study Day 1 and the first date of progression as determined by the Investigator or death (due to any cause). Patients who did not progress and who did not die while on treatment had their event time censored on the last study date on which lack of disease progression was verified. Patients without any recorded tumor response data had their event time censored on study Day 1. Time to death (or overall survival) was defined as the time from the first day of study drug administration to death due to any cause. PFS and overall survival were analyzed based on the modified intent-to-treat (mITT) population, the response evaluable (RE) population and the per protocol (PP) population.

### 2.5 Statistical considerations

The m-ITT safety population included all patients who received at least one dose of L-DOS47. The RE population included all subjects who had measurable disease at baseline, received at least one dose of L-DOS47, and had at least one post-baseline response assessment. The PP population (Phase II only) included all subjects who met the additional requirement of having completed ≥ 4 cycles of L-DOS47 treatment without any major protocol violations.

The Phase II sample size calculation was estimated using the following parameters: a 1-sided test at the significance level of α = 0.05, power of 80%, a null hypothesis of response rate ≤ 1%, and an alternative hypothesis of response rate ≥ 10%. Based on Simon’s optimal two-stage design, seventeen evaluable patients needed to be enrolled in the first stage; if ≥ 1 response(s) out of these initial seventeen patients had been observed, twenty-two additional evaluable patients would have needed to be enrolled.

PK parameters were determined from L-DOS47 concentrations using non-compartmental methods (WinNonlin^®^). Statistical analyses were performed using GraphPad Prism for Windows version 10.1.0 (316). Asterisks in graphs indicate significance levels such that * P < 0.05, ** P < 0.01, *** P < 0.001, **** P < 0.0001.

L-DOS47 concentrations, PK parameters and cytokine levels were summarized using descriptive statistics. Where Kaplan-Meier curves were generated, Log-rank tests were used to make comparisons to investigate whether survival distributions significantly varied amongst groups, and a Bonferroni correction was applied for multiple pairwise comparisons.

Kruskal-Wallis tests were employed for non-parametric pharmacokinetic analyses. Dunn’s tests were used to explore pairwise comparisons in cases where the overall Kruskall-Wallis test was significant. Spearman linear correlations between AUC and each of dose and C_max_ were calculated with associated two-tailed P values. Multiple Wilcoxon tests were applied to investigate the relationship between CEACAM6 and best overall response rate (BORR).

### 2.6 Safety assessments

Abnormal laboratory findings or other assessments judged by the Investigator as clinically significant were recorded as AEs or SAEs. Any medical occurrence reported after informed consent but before treatment start was documented as a non-treatment emergent AE. Events of special interest were defined as any serious unexpected reactions or ≥Grade 3 AEs or SAEs that were possibly, probably, or definitely related to L-DOS47.

### 2.7 Pharmacokinetic and pharmacodynamic assessments

Blood samples were collected from all treated patients to measure L-DOS47 concentrations and test for the presence of ADA, cytokines, and plasma CEACAM6 (schedule in **Supplementary Figure S1**). All patient samples were analyzed using validated methods and under the applicable Good Laboratory Practices (GLP) standards. L-DOS47 plasma concentration was quantified using a sandwich enzyme-linked immunosorbent assay (ELISA) in which sample L-DOS47 was captured with a rabbit polyclonal antibody specific for urease (Rockland Immunochemicals, PA, US) and detected with a peroxidase-conjugated rabbit polyclonal antibody specific for the antibody fragment (generated by Helix BioPharma Corp in house).

The electrochemiluminescence-based ADA assay employed bivalent binding of ADA to both biotin and ruthenium Sulfo-Tag-conjugated L-DOS47, where biotinylated L-DOS47 was the capture antigen on a streptavidin-coated assay plate and ruthenylated L-DOS47 bound to ADA in test samples, forming immune complexes that produced light upon application of an electric potential. This bridging assay format theoretically detects all classes of immunoglobulins, as long as they are functionally multivalent, although it does not distinguish between neutralizing and non-neutralizing antibodies.

Serum cytokines (G-CSF, GM-CSF, IL-1β, IL-1RA, IL-2, IL-4, IL-6, IL-8, IL 10, IL-12, IFNα, IFNγ, IP-10, TNFα) were measured using a multiplex Luminex method based on a human 14-plex custom antibody bead kit (Invitrogen, CA, USA). Only reported values within the validated assay ranges were considered for safety analyses.

CEACAM6 was evaluated using an ELISA that employed a mouse anti-CEACAM6 antibody as the capture antibody and an affinity-purified peroxidase-conjugated goat antibody as the detection antibody.

### 2.8 Immunohistochemistry

Serial sections of the commercially available non-squamous NSCLC tumor microarray (TMA) LC072-01A were purchased from US Biolab (Rockville, MD, USA) and included two cores each from primary tumors of 23 adenocarcinoma cases, plus 8 evaluable cases with both a malignant and matched normal adjacent tumor core, and 4 normal cases in duplicate. Four-micron-thick TMA sections were subjected to 30 minutes of heat-induced epitope retrieval with Tris-EDTA buffer (pH 6.0 for CEACAM6 and pH 9.0 for L-DOS47) on a Dako Omnis autostainer (Agilent Technologies, Santa Clara, CA). The primary antibody for CEACAM6 [EPR23956 (Abcam)] was incubated at a dilution of 1:1000 using a Dako Omnis protocol 20-X-20. The L-DOS47 antibody was incubated at a dilution 1:25 for 45 min. Afterwards, anti-urease antibody (Rockland, dilution 1:100) was added to the slide, followed by the Dako Omnis protocol (30-10R-30) and horseradish peroxidase based 3, 3-diaminobenzidine detection. The interpretation was performed by using a combination of intensity in 4-tiers as absent (0), low (1), moderate (2) and strong (3) and % distribution 0-100%. The PS2+ scoring system was employed for CEACAM6 as expression patterns are reminiscent of those of folate receptor alpha for which this system was developed (38). In this system, the membrane stain intensity is assessed and categorized, where 2 represents moderate staining and 3 represents strong staining; the percentage of tumor cells with moderate and/or strong membrane staining is then calculated compared to the total number of viable tumor cells. Cases were grouped according to the percentage of tumor cells with CEACAM6 staining intensity of ≥2+ as follows: zero to low (absent or less than 50%), medium (≥ 50%) and high (≥ 75%).

## 3 Results

### 3.1 Study population

**Figure 1:**
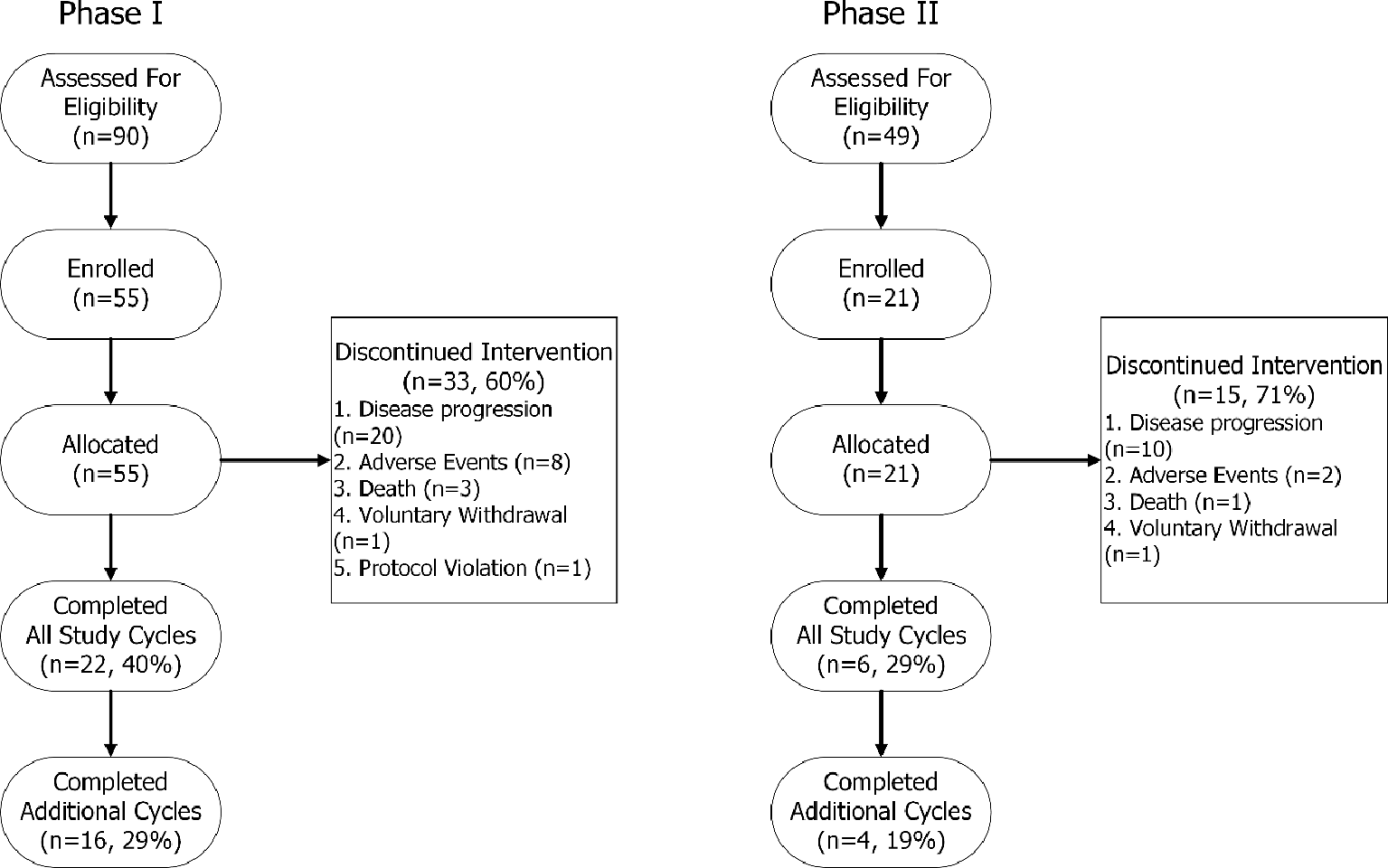
TREND participant flow chart.

Patient demographics and baseline characteristics are summarized in **Table 1**. Ninety patients were screened for Phase I and 49 patients were screened for Phase II (**Figure 1**). Enrolment for Phase I began in October 2012 and the last patient visit for Phase II was in August 2017. The study design is shown in **Supplementary Figure S1**. Of patients who were treated with L-DOS47 in Phase I, 22/55 (40%) completed four treatment cycles as per protocol and 16/55 (29%) continued L-DOS47 treatment for up to 15 cycles. After the last patient enrolled in Phase II, the protocol was amended to limit additional cycles to two due to lack of support for improved outcomes with the intensified dosing regimen. Indeed, 15/21 (71%) patients had discontinued treatment before completing four cycles due to disease progression.

**Table 1:** Patient demographics and baseline characteristics.

Parameters	Phase I		Phase II	
	Mean	Min, Max	Mean	Min, Max
Age (yr)	60.5	33, 83	63.7	44, 74
Weight (kg)	69.2	48, 95	72.7	50, 109

Parameters	Total (N=55)		Total (N=21)	
	N	%	N	%
Male	29	53	11	52
Female	26	47	10	48
Caucasian	55	100	21	100
ECOG				
0	14	25	2	10
1	39	71	18	86
2	2	4	1	5
Histology				
Adenocarcinoma	53	96	21	100
Large Cell Carcinoma	1	2	0	0
Not Otherwise Specified	1	2	0	0
Stage				
IIIA	–	–	1	5
IIIB	7	13	1	5
IV	48	87	19	90
Prior Chemo/Targeted Therapy				
0 Prior Lines	8	15	0	0
1 Prior Line	6	11	6	29
≥ 2 Prior Lines	41	75	15	71
Radiation	26	47	4	19
Surgery	17	31	7	33

### 3.2 Maximum tolerated dose

In Phase I, MTD was not reached at doses up to 13.55 µg/kg in cohort 16. Only one AE (bone pain) met the protocol defined DLT criteria. This AE was observed in cohort 13 (5.76 µg/kg) during infusion on C2D1 and resulted in expansion of this cohort only. As there was only one DLT and L-DOS47 had a short apparent half-life (see section 3.5), the 13.55 µg/kg dose was therefore chosen as the dose for Phase II.

### 3.3 Safety and tolerability

#### 3.3.1 Adverse events

L-DOS47 monotherapy was well tolerated at all dose levels, as evidenced by the extent of patient exposure: 53/55 (96%) Phase I patients were successfully retreated with L-DOS47 and dose interruptions were reported for only 4/422 doses administered (<1%).

**Table 2** summarizes Phase I TEAEs. Forty-four of 55 (80%) patients in the safety population experienced TEAE. The most common TEAEs, reported by 21/55 (38%) patients, were respiratory/thoracic/mediastinal disorders including dyspnea. Of special interest, 15/55 (27%) patients reported dyspnea, but only two dyspnea AEs were assessed as being related to study treatment. Except for one patient (1.8%) who reported the DLT of Grade 4 bone pain and permanently discontinued the drug, all other patients who reported TEAEs resumed dosing following event resolution.

**Table 2:** Phase I: treatment emergent adverse events with overall incidence ≥5%.

Adverse event	Number of patients (%) (n=55)					
	Toxicity grade					
	1	2	3	4	5	Any grade
Dyspnea	5 (9)	7 (13)		2 (4)	1 (2)	15 (27)
Fatigue	4 (7)	4 (7)				8 (15)
Nausea	3 (5)	5 (9)				8 (15)
Cough	4 (7)	2 (4)				6 (11)
Insomnia	3 (5)	3 (5)				6 (11)
Non-small cell lung cancer				1 (2)	5 (9)	6 (11)
Anemia		1 (2)	4 (7)			5 (9)
Asthenia	2 (4)	2 (4)	1 (2)			5 (9)
Decreased Appetite	1 (2)	4 (7)				5 (9)
Vomiting	4 (7)		1 (2)			5 (9)
General physical health deterioration		2 (4)		1 (2)	1 (2)	4 (7)
Hypokalemia	2 (4)	1 (2)	1 (2)			4 (7)
Pneumonia		2 (4)	2 (4)			4 (7)
Abdominal pain	2 (4)	1 (2)				3 (5)
Bone pain		2 (4)		1 (2)		3 (5)
Edema Peripheral	1 (2)	2 (4)				3 (5)
Non-cardiac chest pain	2 (4)	1 (2)				3 (5)

Three patients (5%) experienced infusion reactions consisting of chills, dyspnea and infusion site pain and pruritus, leading to temporary infusion interruptions but which did not result in treatment discontinuation. Most other AEs were also low-grade and resulted only in temporary infusion interruptions. Aside from the DLT, no other significant unexpected toxicity was observed in the first two hours after infusion. There was no clear dose-response relationship between AEs and L-DOS47 dose level as evidenced by the spread of TEAEs across dosing cohorts (**Table 3**).

**Table 3:** Phase I: treatment emergent adverse events across L-DOS47 dosing cohorts with overall incidence ≥5%.

Adverse event	Dose cohorts (µg/kg) (n=55)															
	0.12	0.21	0.33	0.46	0.59	0.78	1.04	1.38	1.84	2.45	3.26	4.33	5.76	7.66	10.19	13.55
Abdominal pain	1 (2)	1 (2)									1 (2)					
Anemia	1 (2)									1 (2)	1 (2)	1 (2)			1 (2)	
Asthenia					1 (2)		1 (2)			1 (2)	1 (2)					1 (2)
Bone pain										1 (2)			1 (2)		1 (2)	
Cough	1 (2)			2 (4)									1 (2)		1 (2)	1 (2)
Decreased appetite						1 (2)	1 (2)			1 (2)	1 (2)				1 (2)	
Dyspnea	2 (4)	1 (2)		1 (2)	2 (4)		2 (4)	1 (2)		1 (2)		1 (2)	2	1 (2)	1 (2)	
Edema peripheral	1 (2)		1 (2)								1 (2)					
Fatigue	1 (2)	1 (2)				2 (4)							2 (4)		2 (4)	
General physical health deterioration	1 (2)	1 (2)						1 (2)			1 (2)					
Insomnia	1 (2)	2 (4)									1 (2)	1 (2)			1 (2)	
Nausea		1 (2)	1 (2)			1 (2)				1 (2)	1 (2)			1 (2)	2 (4)	
Non-cardiac chest pain	1 (2)						2 (4)									
Non-small cell lung cancer								2 (4)	1 (2)	1 (2)	1 (2)					
Pneumonia	1 (2)	1 (2)					1 (2)							1 (2)		
Vomiting						1 (2)				1 (2)	1 (2)				2 (4)	

Other than AV block first degree (2 patients), no TEAEs were reported related to irregular vital signs or electrocardiogram findings. Physical examination, weight, and clinical laboratory values were likewise generally unremarkable.

Twenty-two patients (40%) in the safety population reported SAEs though only anemia (one patient) and bone pain (one patient) were deemed probably related to L-DOS47. The ten deaths (18%) observed in the study were considered unrelated to L-DOS47.

In Phase II, two patients experienced infusion reactions: both had dyspnea and one also reported spinal pain (**Table 4**). These events were assessed as severe but resolved upon drug discontinuation with no additional treatments administered specific to the AEs except allergy medication prescribed to one patient. None of the patients who experienced bone pain at the time of infusion in Phase I or Phase II had known bone metastases.

**Table 4:** Phase II: treatment emergent AEs with overall incidence ≥ 5%.

Adverse event	Number of patients (%) (n=21)					
	Toxicity grade					
	1	2	3	4	5	Any grade
Vomiting	3 (14)	2 (10)	2 (10)			7 (33)
Nausea	3 (14)	2 (10)				5 (24)
Dyspnea			3 (14)			3 (14)
Fatigue	2 (10)	1 (5)				3 (14)
Hyperhidrosis	2 (10)	1 (5)				3 (14)
Asthenia	2 (10)					2 (10)
Constipation	2 (10)					2 (10)
Dyspnea exertional		2 (10)				2 (10)
Edema peripheral	1 (5)	1 (5)				2 (10)
Hypertension	1 (5)	1 (5)				2 (10)
Musculoskeletal chest pain	2 (10)					2 (10)
Non-small cell lung cancer					2 (10)	2 (10)
Pulmonary embolism		1 (5)	1 (5)			2 (10)
Spinal pain		1 (5)	1 (5)			2 (10)
Tachycardia	1 (5)	1 (5)				2 (10)
Abdominal pain	1 (5)					1 (5)
Abdominal pain upper		1 (5)				1 (5)
Anemia		1 (5)				1 (5)
Astrocytoma		1 (5)				1 (5)
Blood albumin decreased		1 (5)				1 (5)
Body temperature increased	1 (5)					1 (5)
Confusional state			1 (5)			1 (5)
Cough		1 (5)				1 (5)
Decreased appetite	1 (5)					1 (5)
Diarrhea		1 (5)				1 (5)
Disease progression					1 (5)	1 (5)
Dyspepsia		1 (5)				1 (5)
Extrapyramidal disorder		1 (5)				1 (5)
Hypotension		1 (5)				1 (5)
Malaise		1 (5)				1 (5)
Myocardial ischemia	1 (5)					1 (5)
Nervousness		1 (5)				1 (5)
Respiratory failure			1 (5)			1 (5)
Sinusitis		1 (5)				1 (5)
Sleep disorder	1 (5)					1 (5)

The most common AEs reported in Phase II for the safety population were gastrointestinal events, experienced by 57% of patients. Of these, nausea and vomiting were most reported. The next-most common AEs were general disorder conditions, particularly fatigue and asthenia (8/21; 38%), and respiratory events (7/21; 33%). Five patients reported dyspnea or exertional dyspnea, with two assessed as related to L-DOS47 treatment.

#### 3.3.2 Laboratory values

Leukocyte counts were reviewed as per CTCAE v4.0. Low grade abnormalities were reported for absolute lymphocyte, monocyte, neutrophil, and/or leukocyte counts in 46/55 patients (84%) but generally did not become more severe during the study; in 24/55 (44%) patients these abnormalities were present at screening. Grade 3 abnormalities were only reported in four patients with G3 lymphopenia, three of whom had had G1-G2 lymphopenia at screening. None of these were classified as AE by the investigator(s). In addition, seven AEs involving disease-typical infections were reported (five pneumonia, one ear infection, one upper respiratory tract infection) and were mostly associated with elevated leukocytes.

Blood ammonia levels were not followed since these L-DOS47 doses were not expected to yield sufficient ammonia to act systemically. Indeed, no neurotoxic AE associated with hyperammonemia were observed. Blood urea nitrogen (BUN) levels remained normal, except for nine patients with elevated BUN at some points on study, although three already had elevated levels at screening.

#### 3.3.3 Cytokines

**Figure 2:**
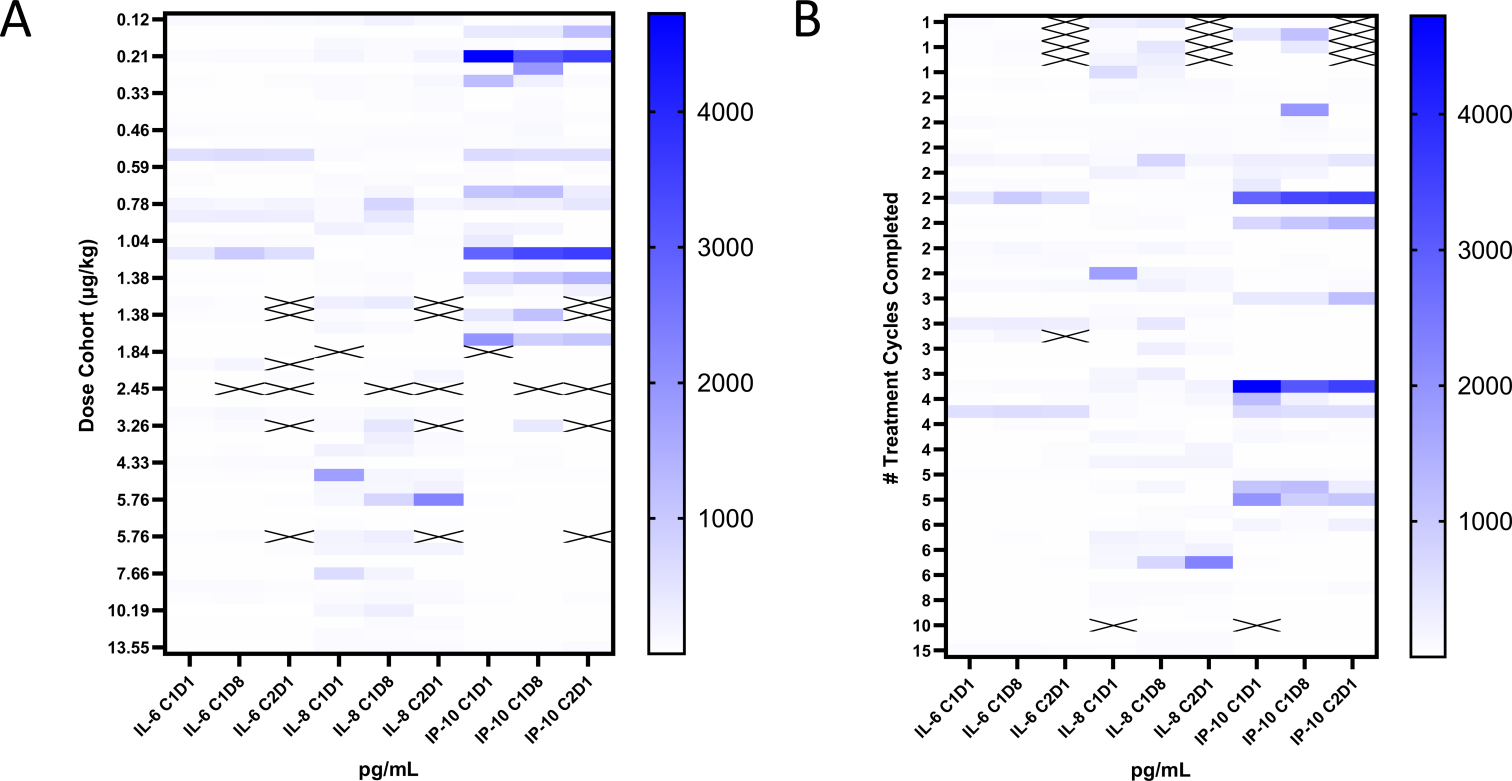
Cytokine responses to L-DOS47 administration. Heat maps of IL-6, IL-8, and IP-10 pre-dose values on C1D1, C1D8, and C2D1 grouped by **(A)** L-DOS47 dose level and **(B)** number of completed treatment cycles.

The protocol called for cytokine monitoring pre-dose to 24 hours post-dose on C1D1 and C1D8 and pre-dose on C2D1. Circulating G-CSF, GM-CSF, IL-2, IL-4, IFNα, and IFNγ remained below the lower limit of quantitation (LLOQ) in all patients at all time points that were sampled and analyzed (**Supplementary Table S1**). While decreases in IL-1β and increases in IL-1RA and IL-12 were seen in a few patients after L-DOS47 administration, these patients also had quantifiable cytokines prior to dosing; changes were relatively minor and transient and did not appear related to dose. Changes were more consistently observed for IL-6, IL-8, and IP-10; however, in many patients these cytokines were quantifiable before L-DOS47 administration (**Figure 2**).

Only one patient with elevated cytokine levels had AEs (chills, elevated temperature, mild dyspnea) temporally associated with L-DOS47 dosing. In additional samples collected for this patient, IL-6 and IL-8 levels were elevated at 1.5 hours, and IL-8 and IP-10 levels were elevated at 6 hours, but all three cytokines were <LLOQ by 24 hours and the AE had resolved. No other signs associated with cytokine release syndrome (CRS) were observed.

### 3.4 Efficacy analysis

The study was not designed to assess efficacy in Phase I. In Phase II, no complete or partial responses were observed in either the RE (n = 19) or PP (n = 5) populations. Testing the 0% response rate against the alternative hypothesis of ≥10% yielded a p-value of P=0.1351 [95% CI: 0-16.82]. The median times to disease progression were 42 days (95% CI: 27 to 75) and 150 days (95% CI:27 to 73 to ∞) for the RE and PP populations respectively. Median times for PFS were the same. Median overall survival was 150 days, 190 days and not yet reached at time data collection was concluded, for safety, RE and PP populations, respectively.

**Figure 3:**
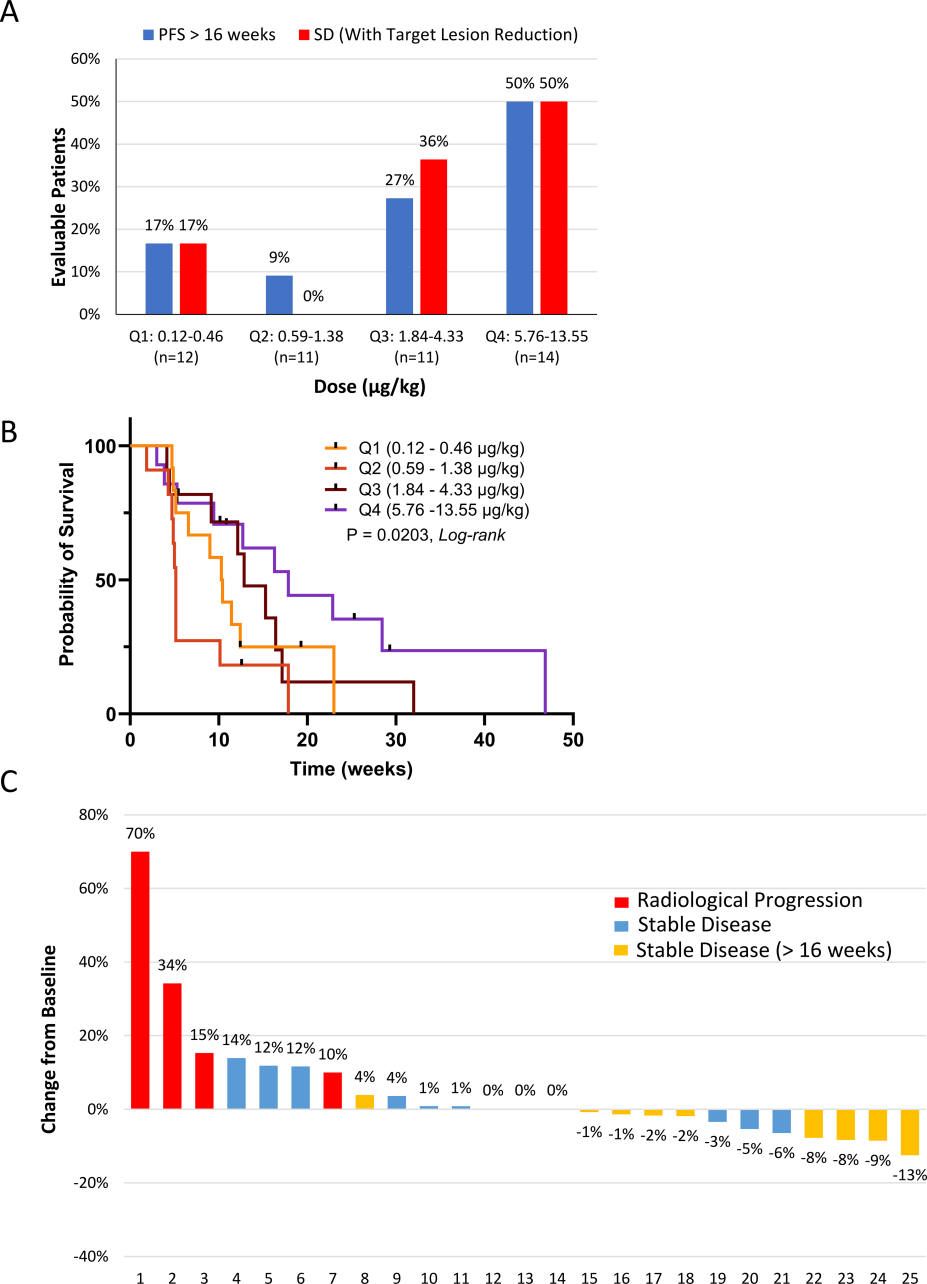
Outcomes in the response-evaluable population (n=48). **(A)** L-DOS47 clinical response. Patients in the 16 dose cohorts were clustered into four quartiles (Q1 – Q4 corresponding to cohorts 1 – 4, 5 – 8, 9 – 12, and 13 – 16) to identify a possible dose-response relationship for progression-free survival (PFS) and stable disease (SD). **(B)** Kaplan-Meier survival curves were generated to investigate PFS (Log-rank P = 0.0203). **(C)** Waterfall plot of target lesion responses in quartiles 3 and 4 (cohorts 9 to 16; n=25). Note that target lesion reduction by ≥8% was seen in four patients experiencing stable disease beyond week 16.

Although no formal efficacy endpoint was specified for Phase I, and there were no CRs or PRs, *post hoc* exploratory analysis was performed on RE patient responses (n=48). Given the large number of dose cohorts and small differences in dose levels between cohorts, the cohorts were grouped into dose quartiles for analysis, at which point a trend towards a positive dose-dependent clinical benefit became evident (**Figure 3A**). In the highest dose quartile, a greater proportion of patients remained progression-free at 16 weeks, had stable disease (SD), and experienced reduction in target lesions. The 16-week cutoff for PFS evaluation translated to approximately 4 months or more than 5 cycles, which was deemed sufficient to distinguish a true treatment effect. A significant difference was identified between the KM curves of the dose quartiles (**Figure 3B**, P=0.0203). Multiple pairwise comparisons of the KM curves were conducted for all four dose quartiles, but prolonged PFS reached statistical significance only for patients in Q4 relative to patients in Q2 (P=0.0069), despite the fact that Q4 was comprised mostly of patients with ≥ 2 previous lines of treatment (**Table 5**, 93%). However, only modest tumor regression (1–13%) was observed in many of the patients in Q3 and Q4 (**Figure 3C**).

**Table 5:** Median PFS by dosing quartile.

Dose (µg/kg)	% Prior lines of therapy			Median PFS (months) [95% Confidence intervals]
	0	1	≥2	
0.12 - 0.46	33.3	8.3	58.3	2.4 [1.1 - ∞]
0.59-1.38	30.8	7.7	61.5	1.2 [1.0 - 2.33]
1.84 - 4.33	0.0	20.0	80.0	3.0 [1.0 - 3.9]
5.76 - 13.55	0.0	6.7	93.3	4.1 [1.2 - ∞]

### 3.5 Pharmacokinetics

**Figure 4:**
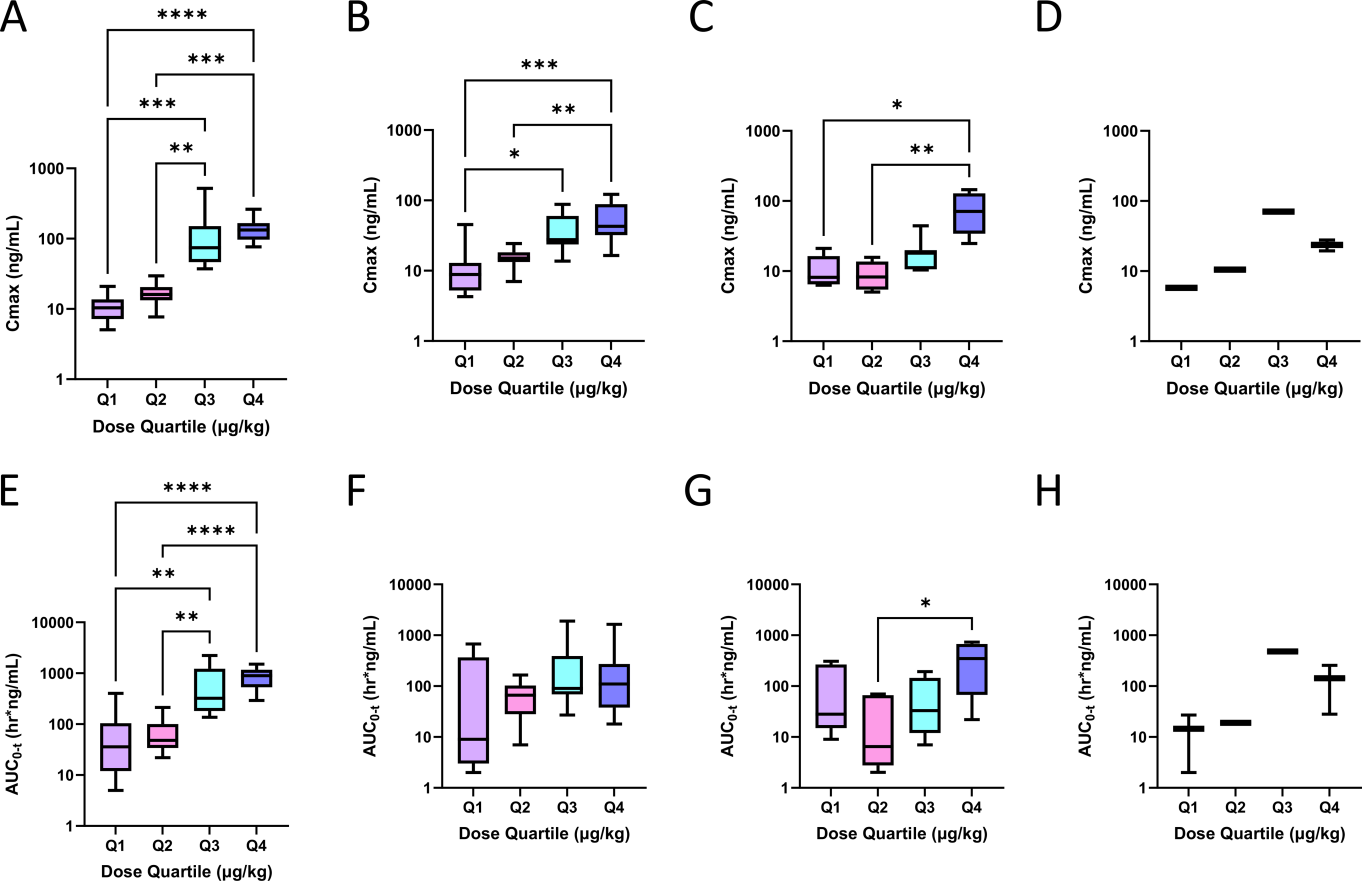
L-DOS47 PK profiles. Grouped by dose quartile, L-DOS47 mean C_max_ and mean area under the curve (AUC) were determined at various timepoints. L-DOS47 mean C_max_ after **(A)** initial (C1D1) dosing and after dosing on **(B)** C1D8, **(C)** C2D8, and **(D)** C4D8; L-DOS47 mean AUC(0-t) after **(E)** initial (C1D1) dosing and after dosing on **(F)** C1D8, **(G)** C2D8 and (H) C4D8. Kruskal-Wallis tests followed by Dunn’s tests for pairwise comparisons were employed in A – H: * P < 0.05, ** P < 0.01, *** P < 0.001, **** P < 0.0001.

After initial dosing in Phase I, mean C_max_ rose with increasing L−DOS47 dose levels (**Figure 4A**), and upon repeat dosing, C_max_ for the higher dose quartiles remained significantly higher than the lower quartiles until C2D8 (**Figures 4B–D**). Likewise, C1D1 AUC_(0-t)_ rose with increasing L−DOS47 dose levels (**Figure 4E**), but apparent AUC_(0-t)_ thereafter was not significantly different other than on C2D8 between Q2 and Q4 (**Figures 4F–H**). Data for individual cohorts are shown in **Supplementary Figure S2**.

T_max_ occurred consistently within the first hour of L-DOS47 infusion in all but two patients. Mean half-life (t_1/2_) on C1D1 where estimable ranged from 3.49-10.9 hours; there was no apparent correlation between t_1/2_ and L-DOS47 dose level (**Supplementary Figure S3**). Mean t_1/2_ upon repeat dosing ranged from 1.09–12 hours where estimable (**Supplementary Table S2**). Similar patterns were observed in Phase II (**Supplementary Table S3**).

**Figure 5:**
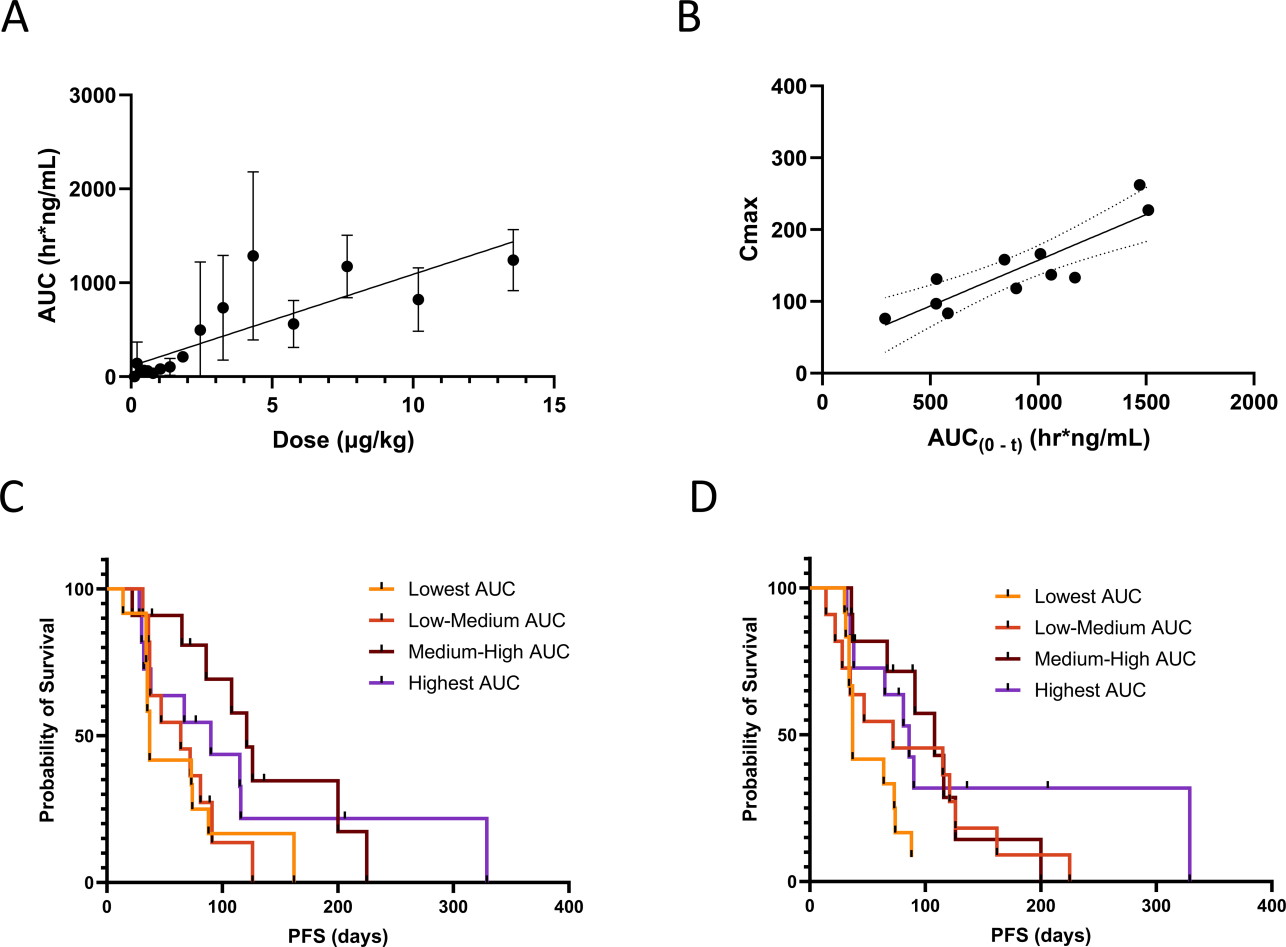
L-DOS47 PK relationship to dose levels and PFS. Spearman correlations were calculated to assess relationships between **(A)** mean AUC_(0-t)_ after initial (C1D1) dosing and dose level (r = 0.8794, two-tailed P < 0.0001) and **(B)** C_max_ and AUC_(0-t)_ in dose quartile 4 after initial (C1D1) dosing (r = 0.8273, two-tailed P = 0.0027); Kaplan-Meier survival curves were generated to investigate progression-free survival (PFS) versus AUC_(0-t)_ after **(C)** initial (C1D1) dosing (Log-rank P = 0.0668) and **(D)** after C1D8 dosing (Log-rank P = 0.0358).

The increase in AUC_(0-t)_ on C1D1 was dose proportional among cohorts, though high inter-subject variability was observed at some doses due to small cohort sizes (**Figure 5A**, r=0.8794, P<0.0001). Furthermore, a strong correlation between C1D1 C_max_ and AUC_(0-t)_ was seen in Q4 (**Figure 5B**, r= 0.8273, P=0.0027). C1D1 systemic exposure indicated a trend towards being predictive of PFS (**Figure 5C**) but was more strongly predictive on C1D8 (**Figure 5D**, P=0.0358).

Despite the apparent decrease in systemic exposure for most subjects after C1D1, three patients showed maintained or increased exposure during subsequent cycles. Furthermore, decreased systemic exposure was also seen in patients who continued L-DOS47 therapy for up to 15 cycles as well as in those with prolonged PFS.

### 3.6 Pharmacodynamics

#### 3.6.1 Immunogenicity

**Figure 6:**
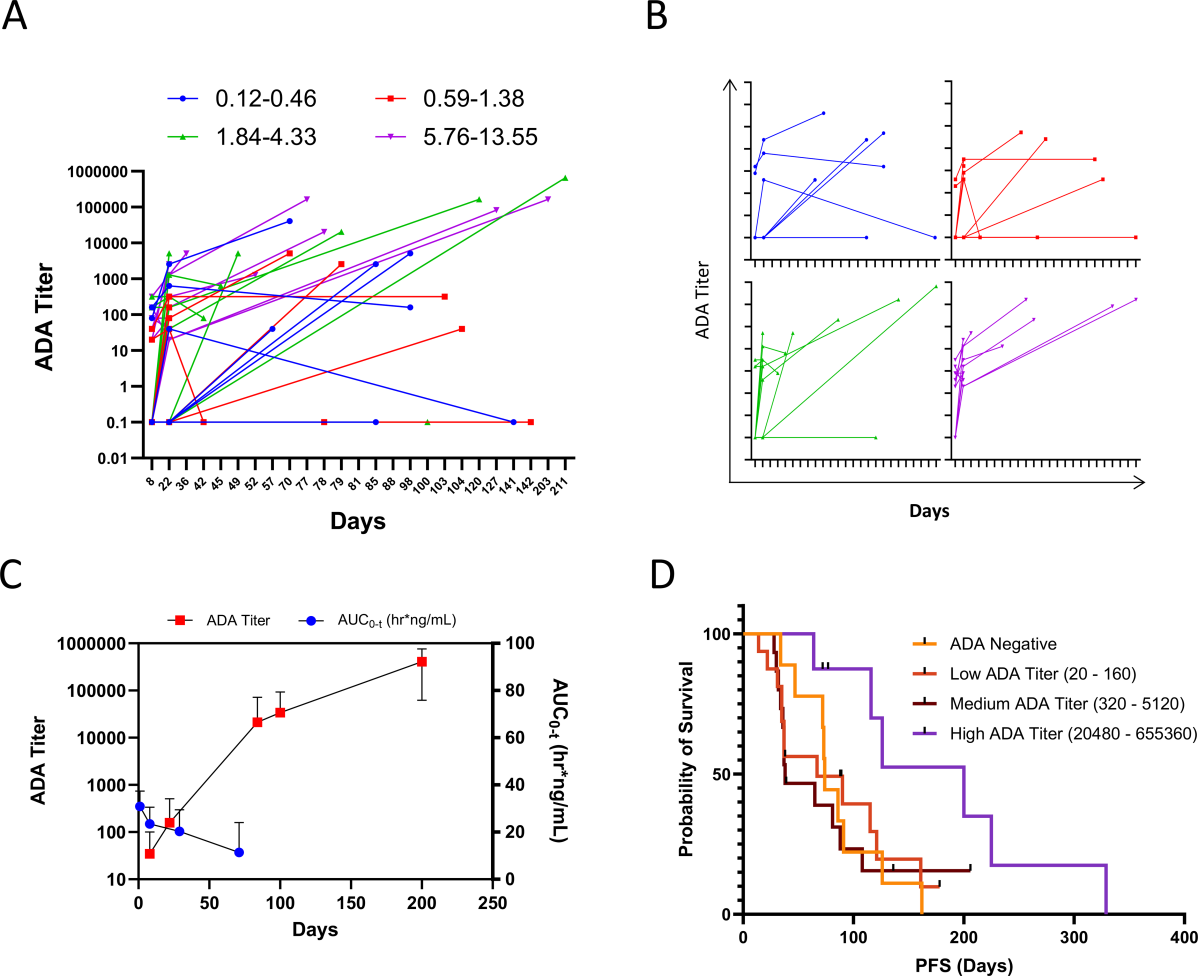
L-DOS47 ADA profiles. **(A)** Individual patient anti-L-DOS47 antibody titers over time; **(B)** ADA titers for individual dose quartiles from panel M over time; **(C)** L-DOS47 AUC_(0-t)_ versus ADA titer over time in 15 patients from the 0.21 – 13.55 µg/kg dose cohorts with complete ADA and PK data sets; **(D)** Kaplan-Meier curve of PFS of ADA-negative versus ADA-positive patients (Log-rank P = 0.1024).

Of Phase 1 patients with ADA data, 43/55 (81%) were ADA positive at some point during treatment. Most titers were low or undetectable at the earliest time point and rose over time (**Figures 6A, B**). While there was no apparent relationship between L-DOS47 dose and ADA titer, patients in the 0.12-2.45 µg/kg cohorts remained ADA negative for longer than those receiving higher doses. Correlating ADA and systemic drug exposure over time in 15 patients for whom data for all protocol-mandated time points was available, AUC_(0-t)_ typically decreased as ADA titer increased (**Figure 6C**). Nevertheless, 14/15 patients achieved SD or SD with modest tumor reduction as their BORR; the fifteenth patient completed five cycles but missed scans to confirm status. Furthermore, no significant difference was observed in PFS for patients who remained ADA-negative throughout the study versus other ADA titer quartiles; in fact, patients with the highest titers tended to have the longest PFS, although this did not reach significance (**Figure 6D**, P=0.1024). A similar correlation between ADA and systemic exposure was observed when all Phase I patients were included (**Supplementary Figure S4A**) and for Phase II (**Supplementary Figure S4B**). Despite the twice-weekly dosing regimen, ADA titers for Phase II (13.55 µg/kg) patients were not significantly different from ADA titers for quartile 4 (5.76-13.55 µg/kg) patients on C1D8 (p >0.9999) and C2D1 (p>0.9999) (**Supplementary Figures S4C, D**).

#### 3.6.2 CEACAM6

Plasma CEACAM6 was quantifiable in only 19/48 (40%) evaluable Phase I patients and ranged from <20 (LLOQ) to 276 ng/mL. Circulating CEACAM6 did not appear to affect L-DOS47 exposure, and in most patients, levels remained relatively stable over time; there was no apparent relationship between plasma CEACAM6 and BORR (**Supplementary Figure S5**). In Phase II patients, CEACAM6 was quantifiable in only 3/24 samples in 13 patients, ranging from 41–154 ng/mL.

### 3.7 Immunohistochemistry

**Figure 7:**
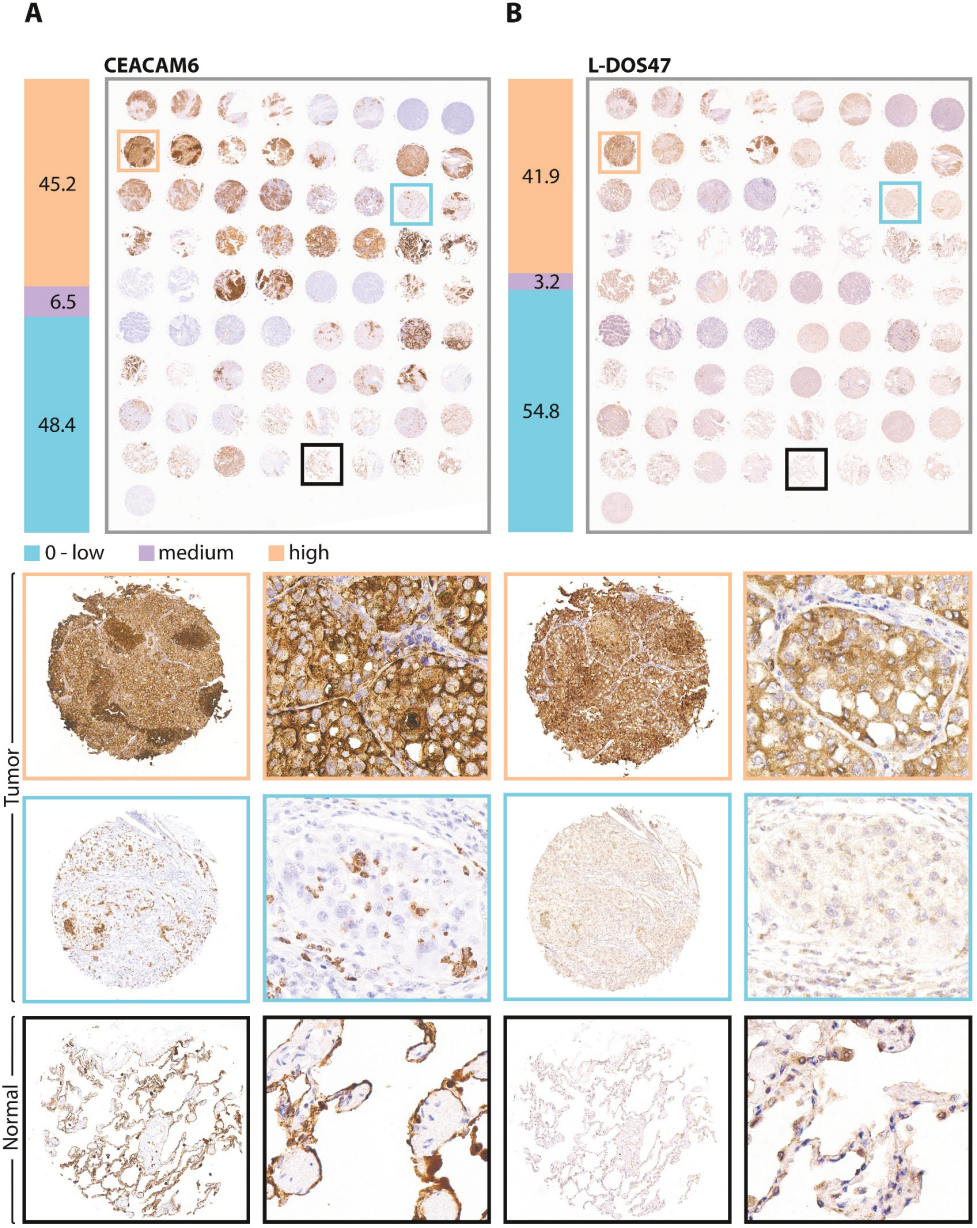
CEACAM6 and L-DOS47 IHC in an unrelated NSCLC cohort. Serial sections of a commercial tissue microarray comprising cores from 31 NSCLC patient cases (from top row: 23 in duplicate plus 9 single cores with matched normal adjacent tissue) and normal tissues (bottom row of duplicate cores from 4 individuals) were assessed by immunohistochemistry for **(A)** CEACAM6, and **(B)** L-DOS47 binding. To the left of each image of the entire tissue microarray, percentages of cases deemed high (orange bar), medium (purple bar) or zero to low (blue bar) expression or binding are indicated. In the panels below, representative images of full tumor cores and higher magnifications thereof are shown; image border colors correspond to scoring results and locations within full tumor microarray. The bottom panels show representative examples of staining in normal tissues (n = 4).

To assess the prevalence of CEACAM6 expression and corresponding L-DOS47 binding in NSCLC, we stained and scored serial sections of a commercially available TMA using the PS2+ scoring system adopted from folate receptor alpha (38). Scoring intensity examples are shown in **Supplementary Figure S6**. High CEACAM6 expression was observed in 45.2% (14/31) of cases, 6.5% (2/31) were medium, and 48.4% (15/31) expressed low to undetectable levels of CEACAM6 (**Figure 7A**). On a serial section of the same TMA, L-DOS47 bound 41.9% (13/31) highly, 3.2% (1/31) moderately, and 54.8% (17/31) exhibited low to no L-DOS47 binding (**Figure 7B**). Good concordance was observed between L-DOS47 binding and CEACAM6 expression (Spearman correlation=0.535, P=0.0019). Of note, normal alveolar lung tissue and alveolar macrophages also exhibited strong membranous CEACAM6 staining (**Figure 7A** bottom panels, black borders). Clinical characteristics of the TMA patient cases are in **Supplementary Table S4**. CEACAM6 expression in normal tissues is reported in **Supplementary Table S5**.

## 4 Discussion

The primary objective of our Phase I study was to determine MTD; however, this was not reached as L-DOS47 monotherapy was well tolerated at doses up to 13.55 µg/kg. Secondary safety endpoints for both phases were achieved, demonstrating overall safety and tolerability of L-DOS47 monotherapy.

Since this was a first-in-human study, it was reassuring that many AEs observed in both trial phases were consistent with expectations for the study population. Non-clinical studies have suggested the potential for neurologic and pulmonary toxicities. However, few neurologic events were reported in the current trial, and none were assessed as related to L-DOS47 treatment. Dyspnea was the most commonly reported respiratory event, though not unusual for this patient population. While 13/15 reports of dyspnea in Phase I and 3/5 in Phase II were assessed as unrelated to L-DOS47 treatment, this remains an adverse event of interest, as potential off-target side effects continue to be monitored. Only a slightly higher incidence of gastrointestinal events (nausea, vomiting) accompanied the intensified dosing frequency in Phase II. No deaths were assessed as related to L-DOS47. Bone pain was observed in 3/55 (5%) patients in Phase I (one a DLT) and 2/21 (9%) patients in Phase II. The AE profile was comparable to that of our Phase I pemetrexed/carboplatin/L-DOS47 combination trial in in NSCLC patients (37). Also, given concerns for the potential of novel biotherapeutics to induce CRS, we monitored cytokine levels in Phase I patients immediately after L-DOS47 administration and found no signs of CRS in any patient at any dose level. No signs of CRS were reported in Phase II of this study or the pemetrexed/carboplatin/L-DOS47 combination trial (37).

In exploratory analyses in Phase I, median PFS in the highest dosing quartile was 4.1 months, which is encouraging as 93% of patients in this quartile had ≥2 lines of prior therapy with a correspondingly poor life expectancy. However, this finding needs to be interpreted cautiously, as this was a single arm study with relatively small patient numbers.

The rationale for amending to twice-weekly dosing in Phase II was arrived at after reviewing available Phase I PFS and PK data, including plasma L-DOS47 concentrations and half-life. Although the Phase I design was not powered to evaluate efficacy, exploratory analyses identified a dose-response trend in PFS at 16 weeks. Since dosing up to 13.55 μg/kg was well tolerated, the intent was to improve the response rate by intensifying the dosing regimen, given the relatively short apparent half-life of L-DOS47. However, since no CR or PR were also observed in Phase II, despite the intensified dosing regimen, development of L-DOS47 as a monotherapy treatment was discontinued. Furthermore, the role of L-DOS47 as a combination treatment to enhance efficacy of other anti-tumor agents was considered a more promising avenue of exploration.

Indeed, our combination trial, which began after initiation of this monotherapy study, reported promising anti-tumor activity (41.7% ORR and 75% clinical benefit) with patients continuing for up to 19 cycles (57 weeks) on L-DOS47 single agent maintenance therapy after chemotherapy ended (37). Furthermore, patients responded at doses well below 13.55 µg/kg, suggesting that the optimal biological dose for L-DOS47 may differ from MTD (39–41) and depend on the combination therapy regimen. Another Phase Ib/II study in advanced pancreatic cancer patients is currently ongoing to evaluate safety and tolerability of escalating doses of L-DOS47 in combination with doxorubicin (NCT04203641). L-DOS47 could also be studied in combination with checkpoint inhibitors (10, 35) or other chemotherapeutics in CEACAM6-expressing tumors.

In addition to the single arm design with limited patient numbers, a major limitation of the current study is that patient tumors were not screened for CEACAM6 expression prior to study enrollment, so baseline CEACAM6 expression was unknown. Additionally, it could not be determined in this study whether plasma CEACAM6 levels were a surrogate for tumor CEACAM6 expression or a biomarker for efficacy. To address this, we recently developed a clinically translatable CEACAM6 IHC protocol, which we verified by assessing unrelated NSCLC cases. As less than half of those cases expressed appreciable CEACAM6 levels and since CEACAM6 expression was not an inclusion criterion in our trial, patient heterogeneity in tumor CEACAM6 may have explained the modest efficacy we observed, since without sufficient CEACAM6, L-DOS47 could not bind and alkalinize the TME.

Moving forward, medium to high CEACAM6 expression will be an eligibility criterion for future L-DOS47 trials. Development of this IHC method into a companion diagnostic for CEACAM6 expression in patient samples would involve evaluation of concordance between results obtained with the commercial anti-CEACAM6 antibody and the anti-CEACAM6 nanobody component of L-DOS47. Additionally, the suitability of the scoring system used in the current study for the method’s intended clinical use would be verified in order to identify the patient population with sufficient levels of CEACAM6 expression to benefit from L-DOS47 treatment.

Lack of data regarding the tumor mutational status for each patient was an additional limitation. Unfortunately, at the time this study was conducted, it was not standard practice to determine mutational status and no historical biopsy tissues could be retrieved for this purpose. However, since the proposed L-DOS47 mechanism of action does not target specific tumor mutations, the impact of tumor mutational status should be negligible.

While tumor urea levels were not measured in this study, blood urea nitrogen levels for most patients fell within the normal range (3.57-17.85 mM), which is in the physiologically relevant range for the reported K_m_ for jack bean urease of 2–3 mM (42). CEST-MRI imaging has been used in animal models to show that L-DOS47 reduces the TME pH of acidic tumors (35), which indirectly demonstrates that sufficient urea is present in the TME for L-DOS47 to achieve its mechanism of action. Direct assessment of TME pH remains a limitation in clinical settings, as CEST-MRI is not yet in widespread use clinically, especially for non-brain tumors (43). For future clinical trials, potential surrogates for direct pH measurement under consideration include FDG-PET and other specialized PET scans to assess CD8+ T-cell tumor infiltration as a measure of immune system reactivation.

Given the intended delivery of L-DOS47 to CEACAM6-expressing tumors and that urease activity would be localized to the TME (25), systemic ammonia should not increase appreciably at the L-DOS47 doses investigated here; indeed, no neurotoxic signs related to hyperammonemia were reported. Furthermore, in the event of sustained excess urease activity due to L-DOS47, routinely monitored pre-dose BUN levels would have been expected to decrease; this was not observed in any patients (44).

Virtually all patients developed treatment-emergent ADA that generally corresponded to apparently reduced L-DOS47 plasma levels, which was not unexpected given that the urease component of L-DOS47 is plant-derived. However, antibodies appeared in some patients whose L-DOS47 exposure did not consistently decline, and others experienced a decline in L-DOS47 exposure without increased titers. Since our assay was not specific for neutralizing antibodies, the impact of ADA on systemic exposure to L-DOS47 could not be conclusively determined. The antibody responses were likely not predominantly neutralizing since longest PFS tended to be seen in the highest titer group. These results are consistent with those from our combination trial, in which patients with early progressive disease remained ADA-negative or had very low titers while those who achieved best ORR tended to have the highest ADA titers (37). Future trials will monitor total ADA and also evaluate ADA that neutralize either urease enzyme activity or CEACAM6 binding, either of which could potentially interfere with efficacy.

## 5 Conclusion

In summary, L-DOS47 was well tolerated as a monotherapy at doses up to and including 13.55 μg/kg but did not result in any CRs or PRs. While the data does not support L-DOS47 as a monotherapy, *post hoc* exploratory analyses identified a trend at higher L-DOS47 doses that were associated with PFS ≥16 weeks with additional maintenance cycles completed. This, as well as the likelihood that many may have lacked appreciable tumor CEACAM6 expression, may provide direction for design of future L-DOS47 combination studies. In future, patient screening for CEACAM6 positive tumors by IHC has the potential to help identify patients most likely to benefit from L-DOS47 therapy.

## Data Availability

The datasets presented in this article are not readily available because requests need to be submitted directly to Helix BioPharma Corp. Requests to access the datasets should be directed to corporate@helixbiopharma.com
