# Supplementary material for: Safety of unconventional antibody-drug conjugate L-DOS47 in a phase I/II monotherapy study targeting advanced NSCLC
Source: Front Oncol. 2025 Aug 11;15:1544967. doi: 10.3389/fonc.2025.1544967 (PMC12375440; doi:10.3389/fonc.2025.1544967)
Supplement: Supplementary file 1 [file DataSheet1.docx]

**Safety of unconventional antibody-drug conjugate L-DOS47 in a Phase I/II monotherapy study targeting advanced NSCLC**

**SUPPLEMENTARY MATERIAL**

**Supplementary Tables:**

**Table S1.** Summary of Cytokine Results for Phase I Patients (Safety Analysis)

**Table S2.** L-DOS47 Summary (Mean ± STD) Pharmacokinetic Parameters (Phase I)

**Table S3.** L-DOS47 Summary (Mean ± STD) Pharmacokinetic Parameters (Phase II)

**Table S4.** NSCLC TMA Cases - Patient Characteristics

**Table S5.** CEACAM6 Expression in Normal Tissues.

**Supplementary Figures**

**Figure S1.** Study schematic for (*A*) Phase I and (*B*) Phase II.

**Figure S2.** L-DOS47 mean C_max_ and AUC_(0-t)_ on C1D1, C1D8, C2D8 and C4D8.

**Figure S3.** Relationship between L-DOS47 half-life and dose.

**Figure S4.** L-DOS47 immunogenicity.

**Figure S5.** Relationship between plasma CEACAM6 and BORR.

**Figure S6.** Scoring intensity examples for CEACAM6 immunohistochemistry staining.

**Supplementary Tables:**

**Table S1.** Summary of Cytokine Results for Phase I Patients (Safety Analysis)

| Cytokine | Cycle/Day | Evaluable Patients | Predose >LLOQ | % | Predose <LLOQ | % | All below LLOQ |
| --- | --- | --- | --- | --- | --- | --- | --- |
| G-CSF | All CD | 52 | 0 | 0.0 | 52 | 100.0 | 52 |
| GM-CSF | All CD | 52 | 0 | 0.0 | 52 | 100.0 | 52 |
| IL-1β | C1D1 | 52 | 3 | 5.8 | 0 | 0.0 | 49 |
|  | C1D8 | 51 | 1 | 2.0 | 0 | 0.0 | 50 |
|  | C2D1 | 47 | 2 | 4.3 | 0 | 0.0 | 45 |
| IL-1RA | C1D1 | 52 | 1 | 1.9 | 1 | 1.9 | 50 |
|  | C1D8 | 51 | 1 | 2.0 | 1 | 2.0 | 49 |
|  | C2D1 | 47 | 1 | 2.1 | 0 | 0.0 | 46 |
| IL-2 | All CD | 52 | 0 | 0.0 | 0 | 0.0 | 52 |
| IL-4 | All CD | 52 | 0 | 0.0 | 0 | 0.0 | 52 |
| IL-6 | C1D1 | 52 | 15 | 28.8 | 15 | 52.0 | 22 |
|  | C1D8 | 51 | 17 | 33.3 | 14 | 42.0 | 20 |
|  | C2D1 | 47 | 17 | 36.2 | 1 | 2.8 | 29 |
| IL-8 | C1D1 | 52 | 21 | 40.4 | 9 | 22.3 | 32 |
|  | C1D8 | 51 | 20 | 39.2 | 15 | 38.3 | 16 |
|  | C2D1 | 47 | 16 | 34.0 | 1 | 2.1 | 30 |
| IL-10 | C1D1 | 52 | 0 | 0.0 | 1 | 1.9 | 51 |
|  | C1D8 | 51 | 0 | 0.0 | 1 | 2.0 | 50 |
|  | C2D1 | 47 | 0 | 0.0 | 1 | 2.1 | 46 |
| IL-12 | C1D1 | 52 | 1 | 1.9 | 3 | 5.8 | 48 |
|  | C1D8 | 51 | 1 | 2.0 | 3 | 5.9 | 48 |
|  | C2D1 | 47 | 1 | 2.1 | 2 | 4.3 | 44 |
| IFN-α | All CD | 52 | 0 | 0.0 | 0 | 0.0 | 52 |
| IFN-γ | All CD | 52 | 0 | 0.0 | 0 | 0.0 | 52 |
| IP-10 | C1D1 | 52 | 11 | 21.2 | 3 | 14.2 | 39 |
|  | C1D8 | 51 | 10 | 19.6 | 6 | 30.6 | 35 |
|  | C2D1 | 47 | 9 | 19.1 | 1 | 5.2 | 38 |
| TNFα | C1D1 | 52 | 0 | 0.0 | 1 | 1.9 | 51 |
|  | C1D8 | 51 | 0 | 0.0 | 1 | 2.0 | 50 |
|  | C2D1 | 47 | 0 | 0.0 | 1 | 2.1 | 46 |

**Table S2.** L-DOS47 Summary (Mean ± STD) Pharmacokinetic Parameters (Phase I)

| Dose  (µg/kg) | Cycle/  Day | T_max_^1^  (hr) | C_max_  (ng/mL) | Tlast^1^  (hr) | AUC_(0 - t)_  (hr*ng/  mL) | AUC_(0 - 24)_  (hr*ng/  mL) | T_1/2_  (hr) | CL  (mL/hr/kg) | V_ss_  (mL/kg) |
| --- | --- | --- | --- | --- | --- | --- | --- | --- | --- |
| 0.12 | 1/1 | NC | NC | NC | NC | NC | NC | NC | NC |
|  | 1/8 | NC | NC | NC | NC | NC | NC | NC | NC |
|  | 2/8 | NC | NC | NC | NC | NC | NC | NC | NC |
| 0.21 | 1/1 | 0.67  (0.5 - 0.83) | 6.47 ±  1.80 | 3 (1.5 - 72) | 140 ±  228 | 55.3 ± 78.0 | NC | NC | NA |
|  | 1/8 | 0.46  (0.25 - 0.67) | 6.56 ±  ID | 36.4  (0.833-72) | 185 ±  ID | 64.2 ±  ID | NC | NC | NC |
|  | 2/8 | 0.67  (0.5 - 4) | 9.52 ±  4.59 | 3 (1.5-24) | 92.7 ±  139 | 94.1 ±  138 | NC | NC | NC |
|  | 4/8 | 0.67  (0.67 - 0.67) | 5.89 ±  ID | 6 (6 – 6) | 27.3 ±  ID | 33.5 ±  ID | NC | NC | NC |
| 0.33 | 1/1 | 0.5  (0.5 - 0.67) | 13.9 ±  6.23 | 6 (2-12) | 54.2 ± 54.1 | 65.6 ± 65.6 | 4.74 ±  ID | 2.31 ±  ID | NA |
|  | 1/8 | 0.58  (0.5 - 0.67) | 6.06 ±  ID | 1.5 (1-2) | 5.80 ±  ID | 7.49 ±  ID | NC | NC | NC |
|  | 2/8 | 0.67  (0.67 - 0.67) | 6.30 ±  ID | 4 (4-4) | 19.0 ±  ID | 23.1 ±  ID | NC | NC | NC |
|  | 4/8 | 0.5  (0.5 - 0.5) | 5.51 ±  ID | 0.833  (0.833-0.833) | 2.34 ±  ID | 2.76 ±  ID | NC | NC | NC |
| 0.46 | 1/1 | 0.67  (0.5 - 0.83) | 13.6 ± 0.179 | 12 (3-12) | 66.7 ± 38.3 | 90.2 ± 57.7 | NC | NC | NA |
|  | 1/8 | 1  (0.5 - 1.5) | 22.9 ±  19.6 | 9 (1-48) | 249 ±  370 | 165 ±  220 | NC | NC | NC |
|  | 2/8 | 0.58  (0.5 - 0.67) | 15.1 ±  ID | 15 (6-24) | 173 ±  ID | 177 ±  ID | NC | NC | NC |
|  | 4/8 | NC | NC | NC | NC | NC | NC | NC | NC |
| 0.59 | 1/1 | 0.67  (0.67 - 0.83) | 15.1 ±  4.95 | 6 (6-12) | 62.2 ± 20.3 | 86.6 ± 49.8 | NC | NC | NA |
|  | 1/8 | 0.5  (0.5 - 0.67) | 12.6 ±  5.22 | 3 (1.5-12) | 41.6 ± 42.5 | 55.2 ± 61.7 | NC | NC | NC |
|  | 2/8 | 0.83  (0.83 - 0.83) | 13.0 ±  ID | 9 (9-9) | 64.6 ±  ID | 71.1 ±  ID | NC | NC | NC |
|  | 4/8 | 0.67  (0.67 - 0.67) | 10.5 ±  ID | 3 (3-3) | 18.6 ±  ID | 21.0 ±  ID | NC | NC | NC |
| 0.78 | 1/1 | 0.5  (0.5 - 0.5) | 14.9 ±  1.19 | 3 (3-6) | 35.2 ± 10.5 | 39.7 ± 12.4 | NC | NC | NA |
|  | 1/8 | 0.75  (0.67 - 0.83) | 14.2 ±  ID | 16.5 (9-24) | 115 ±  ID | 118 ±  ID | NC | NC | NC |
|  | 2/8 | 0.58  (0.5 - 0.67) | 10.9 ±  ID | 5 (1-9) | 36.6 ±  ID | 44.9 ±  ID | NC | NC | NC |
| 1.04 | 1/1 | 0.67  (0.5 - 0.67) | 21.2 ±  2.95 | 9 (4-12) | 80.6 ± 33.9 | 94.6 ± 44.2 | NC | NC | NA |
|  | 1/8 | 0.54  (0.25 - 0.83) | 18.9 ±  ID | 10.5 (9-12) | 116 ±  ID | 141 ±  ID | NC | NC | NC |
|  | 2/8 | 0.5  (0.5 - 0.5) | 8.01 ±  ID | 1.25 (1-1.5) | 6.57 ±  ID | 7.71 ±  ID | NC | NC | NC |
| 1.38 | 1/1 | 0.5  (0.25 - 0.83) | 17.5 ±  9.07 | 8 (4-24) | 104 ±  91.1 | 114 ±  93.8 | 4.00 ±  ID | 7.92 ±  ID | NA |
|  | 1/8 | 0.83  (0.67 - 0.83) | 17.7 ±  5.85 | 4 (3-6) | 43.2 ± 22.3 | 48.6 ± 25.5 | NC | NC | NC |
|  | 2/8 | 0.83  (0.83 - 0.83) | 5.03 ± ID | 1 (1-1) | 2.02 ±  ID | 3.12 ±  ID | NC | NC | NC |
|  | 4/8 | NC | NC | NC | NC | NC | NC | NC | NC |
| 1.84 | 1/1 | 0.5  (0.5 - 0.67) | 47.0 ±  2.29 | 11.8 (11.8-12) | 210 ±  21.6 | 262 ±  34.2 | 4.59 ±  ID | 6.94 ±  ID | NA |
|  | 1/8 | 0.67  (0.5 - 0.83) | 32.1 ±  9.69 | 9 (3-11.3) | 114 ±  92.1 | 130 ±  111 | 4.08 ±  ID | 7.25 ±  ID | 22.2 ±  ID |
|  | 2/8 | 0.58  (0.5 - 0.67) | 27.9 ± ID | 6.5 (1-12) | 100 ±  ID | 118 ±  ID | 4.80 ±  ID | 8.06 ±  ID | 72.9 ±  ID |
|  | 4/8 | 0.67  (0.67 - 0.67) | 70.6 ± ID | 24 (24-24) | 479 ±  ID | 479 ± ID | 9.31 ±  ID | 3.29 ±  ID | 37.2 ±  ID |
| 2.45 | 1/1 | 0.67  (0.5 - 0.83) | 78.6 ±  64.8 | 9 (9-72) | 497 ±  722 | 352 ±  343 | 3.49 ±  ID | 14.5 ±  ID | NA |
|  | 1/8 | 0.5  (0.5 - 1) | 33.6 ±  20.3 | 9 (6-72) | 322 ±  472 | 204 ±  206 | 2.38 ±  ID | 20.8 ±  ID | 475 ±  ID |
|  | 2/8 | 0.58  (0.5 - 0.67) | 18.6 ±  ID | 2 (1-3) | 25.8 ±  ID | 30.4 ±  ID | NC | NC | NC |
|  | 4/8 | NC | NC | NC | NC | NC | NC | NC | NC |
| 3.26 | 1/1 | 0.5  (0.5 - 0.67) | 96.7 ±  42.7 | 12 (11.5-72) | 734 ±  559 | 665 ±  330 | 7.77 ±  5.63 | 5.26 ± 2.71 | NA |
|  | 1/8 | 0.58  (0.5 - 0.67) | 48.4 ±  ID | 10.7 (8.67-12.8) | 241 ±  ID | 279 ±  ID | 4.85 ±  ID | 7.10 ±  ID | 82.3 ±  ID |
|  | 2/8 | 0.5  (0.5 - 0.5) | 19.9 ±  ID | 3 (3-3) | 33.4 ±  ID | 36.8 ±  ID | NC | NC | NC |
|  | 4/8 | NC | NC | NC | NC | NC | NC | NC | NC |
| 4.33 | 1/1 | 0.5  (0.42 - 0.67) | 137 ±  29.3 | 48 (12-72) | 1290 ± 898 | 909 ±  350 | 10.9 ±  ID | 5.78 ±  ID | NA |
|  | 1/8 | 0.67  (0.5 - 0.83) | 55.8 ±  ID | 37 (2-72) | 961 ±  ID | 447 ±  ID | NC | NC | NC |
|  | 2/8 | 0.58  (0.5 - 0.67) | 10.5 ±  ID | 13 (2-24) | 79.9 ±  ID | 81.5 ±  ID | NC | NC | NC |
| 5.76 | 1/1 | 0.67  (0.5 - 0.67) | 105 ±  23.9 | 18 (12-36) | 561 ±  251 | 560 ±  181 | 6.68 ±  3.41 | 10.6 ± 4.67 | NA |
|  | 1/8 | 0.5  (0.5 - 0.67) | 31.9 ±  17.6 | 3.5 (2-6) | 52.1 ± 39.0 | 56.6 ± 41.2 | 1.98 ±  ID | 50.4 ±  ID | 620 ±  ID |
|  | 2/8 | 0.5  (0.25 - 0.5) | 55.7 ±  28.3 | 9 (1.5-24) | 320 ±  370 | 325 ±  368 | 3.79 ±  ID | 26.6 ±  ID | 771 ±  ID |
|  | 4/8 | 1.05  (1.05 - 1.05) | 27.8 ±  ID | 1.05 (1.05-1.05) | 27.6 ±  ID | 40.8 ±  ID | NC | NC | NC |
| 7.66 | 1/1 | 0.67  (0.67 - 0.83) | 173 ±  48.9 | 35.3 (24-36) | 1170 ±  332 | 1090 ±  256 | 8.31 ±  2.30 | 6.51 ±  1.96 | NA |
|  | 1/8 | 0.5  (0.5 - 0.5) | 54.4 ±  29.8 | 9 (6-11.5) | 148 ±  41.9 | 169 ±  60.5 | 3.75 ± 0.916 | 49.3 ± 16.5 | 1100 ± 731 |
|  | 2/8 | 0.5  (0.5 - 0.5) | 145 ±  ID | 12 (12-12) | 493 ±  ID | 571 ±  ID | 5.62 ±  ID | 13.4 ±  ID | 168 ±  ID |
|  | 4/8 | NC | NC | N | NC | NC | NC | NC | NC |
| 10.19 | 1/1 | 0.5  (0.5 - 0.5) | 137 ±  ID | 36 (36-36) | 1060 ±  ID | 963 ±  ID | 8.53 ±  ID | 9.20 ±  ID | NA |
|  | 1/8 | 0.62  (0.57 - 0.67) | 56.9 ±  ID | 7.5 (3-12) | 203 ±  ID | 268 ±  ID | 1.09 ±  ID | 252 ±  ID | 4820 ±  ID |
|  | 2/8 | NC | NC | NC | NC | NC | NC | NC | NC |
|  | 4/8 | NC | NC | NC | NC | NC | NC | NC | NC |
| 13.55 | 1/1 | 0.75  (0.67 - 0.83) | 214 ±  ID | 36 (36-36) | 1240 ±  ID | 1150 ±  ID | 9.71 ±  ID | 10.7 ±  ID | NA |
|  | 1/8 | 0.58  (0.5 - 0.67) | 112 ±  ID | 30 (12-48) | 957 ±  ID | 790 ±  ID | 12.0 ±  ID | 7.92 ±  ID | 146 ±  ID |
|  | 2/8 | NC | NC | NC | NC | NC | NC | NC | NC |
|  | 4/8 | 24  (24 - 24) | 19.4 ±  ID | 24 (24-24) | 257 ±  ID | 257 ±  ID | NC | NC | NC |
| STD = Standard deviation; ID = Insufficient data; NC = Not calculated; ^1^Median (Min – Max)  T_max_, time after dosing at which the maximum concentration was observed; C_max_, maximum observed concentration measured after dosing; T_last_, time of last measurable (positive) concentration; AUC_(0-t)_, area under the concentration versus time curve from the start of dose administration to the time after dosing at which the last quantifiable concentration was observed; AUC_(0-24)_, area under the concentration versus time curve from the start of dose administration to 24 hours post dose; T_1/2_, apparent terminal elimination half life; Cl, apparent clearance rate of drug from the analyzed matrix; V_ss_, apparent volume of distribution of the drug in the system at steady state. | | | | | | | | | |

| Cycle/  Day | T_max_^1^  (hr) | C_max_  (ng/mL) | T_last_^1^  (hr) | AUC_(0 - t)_  (hr*ng/mL) | AUC_(0 - 24)_  (hr*ng/mL) | T_1/2_  (hr) | CL  (mL/hr/kg) | V_ss_  (mL/kg) |
| --- | --- | --- | --- | --- | --- | --- | --- | --- |
| 1/4 | 0.67  (0.5 - 1) | 260 ± 98.2 | 48  (10.9 - 72) | 2090 ± 1190 | 1790 ± 819 | 10.2 ± 5.49 | 8.94 ± 6.67 | 86.2 ± 41.4 |
| 1/8 | 0.5  (0.45 - 0.67) | 134 ± 224 | 12  (1 - 72) | 475 ± 435 | 399 ± 324 | 3.97 ± 2.66 | 162 ± 318 | 937 ± 1710 |
| 1/11 | 0.5  (0.45 - 0.67) | 55.7 ± 41.4 | 24  (1.5 - 72) | 408 ± 419 | 324 ± 315 | 11.9 ± 7.32 | 40.3 ± 39.0 | 396 ± 149 |
| 2/4 | 0.5  (0.43 - 0.67) | 172 ± 325 | 12  (0.833 - 72) | 401 ± 474 | 326 ± 351 | 7.05 ± 7.88 | 61.9 ± 84.7 | 243 ± 163 |
| 2/8 | 0.5  (0.5 - 48) | 48.6 ± 93.6 | 24.75  (1 - 72) | 347 ± 510 | 230 ± 435 | 19.4 ± ID | 9.80 ± ID | 152 ± ID |
| 2/11 | 0.5  (0.5 - 36) | 80.2 ± 139 | 42  (2 - 72) | 509 ± 590 | 509 ± 891 | NC | NC | NC |
| STD = Standard deviation; ID = Insufficient data; NC = Not calculated; ^1^Median (Min – Max)  T_max_, time after dosing at which the maximum concentration was observed; C_max_, maximum observed concentration measured after dosing; T_last_, time of last measurable (positive) concentration; AUC_(0-t)_, area under the concentration versus time curve from the start of dose administration to the time after dosing at which the last quantifiable concentration was observed; AUC_(0-24)_, area under the concentration versus time curve from the start of dose administration to 24 hours post dose; T_1/2_, apparent terminal elimination half life; Cl, apparent clearance rate of drug from the analyzed matrix; V_ss_, apparent volume of distribution of the drug in the system at steady state. | | | | | | | | |

**Table S3.** L-DOS47 Summary (Mean ± STD) Pharmacokinetic Parameters (Phase II)

**Table S4.** NSCLC TMA Cases - Patient Characteristics

| **Characteristic** | **n (of 31)** | **%** |
| --- | --- | --- |
| **Age** |  |  |
| 41-50 | 1 | 3 |
| 51-60 | 12 | 39 |
| 61-70 | 11 | 35 |
| >70 | 7 | 23 |
| **Sex** |  |  |
| F | 14 | 45 |
| M | 17 | 55 |
| **Pathology** |  |  |
| Grade 1 | 2 | 6 |
| Grade 2 | 24 | 77 |
| Grade 3 | 5 | 16 |
| **Stage** |  |  |
| 1A | 1 | 3 |
| 1A2 | 5 | 16 |
| 1A3 | 8 | 26 |
| 1B | 1 | 3 |
| IIA | 2 | 6 |
| IIB | 4 | 13 |
| IIIA | 8 | 26 |
| IIIB | 2 | 6 |

**Table S5.** CEACAM6 Expression in Normal Tissues.

|  | **CEACAM6 Expression Pattern** |
| --- | --- |
| **High expressors** |  |
| BxPC3 tumor (positive control) | 100% membranous, stronger than cytoplasmic |
| Lung alveolar epithelium | 100% strong membranous |
| Macrophages | 100% strong cytoplasmic |
| Mature squamous epithelium | 100% strong |
| Appendix | Strong on surface, moderate in crypts, positive in neutrophils |
|  |  |
| **Low expressors** |  |
| Colon | Moderate on surface |
| Lung small bronchi | Weak focal dotted |
| Mature squamous epithelium | Weak to moderate in intermediate layer |
|  |  |
| **Negative** |  |
| Endometrial stroma | Absent |
| Endometrium | Absent |
| Fallopian tube | Absent |
| Kidney | Absent |
| Liver | Absent |
| Lymphoid tissue (tonsil, spleen) | Absent |
| Myometrium | Absent |
| Pancreas | Absent |
| Placenta | Absent |
| Spleen | Absent in lymphoid cells, strong in macrophages |
| Stomach | Absent |
| Testis | Absent |

**Supplementary Figures**

A

**Quartile 1**

0.12 μg/kg

0.21 μg/kg

0.33 μg/kg

0.46 μg/kg

**Quartile 2**

0.59 μg/kg

0.78 μg/kg

1.04 μg/kg

1.38 μg/kg

**Quartile 3**

1.84 μg/kg

2.45 μg/kg

3.26 μg/kg

4.33 μg/kg

**Quartile 4**

5.76 μg/kg

7.66 μg/kg

10.19 μg/kg

13.55 μg/kg

B

| **L-DOS47 DOSING: D1, D4, D8, D11 OF 21 DAY CYCLE (PHASE II)** | | |
| --- | --- | --- |
| **Assay** | **Cycles/Days** | **Time Points** |
| PK | C1D1, C2D1 | 0, 30m |
|  | C1D4, C1D8, C1D11  C2D4, C2D8, C2D11 | 0, 30, 40, 50m, 1, 1.5, 2, 4, 6 , 9, 12, 24, 36, 48, 72h |
| ADA | C1D8, C2D1, EOT, FUP | 0 |
| CEACAM6 | C2D1, C4D11/EOT | 0 |


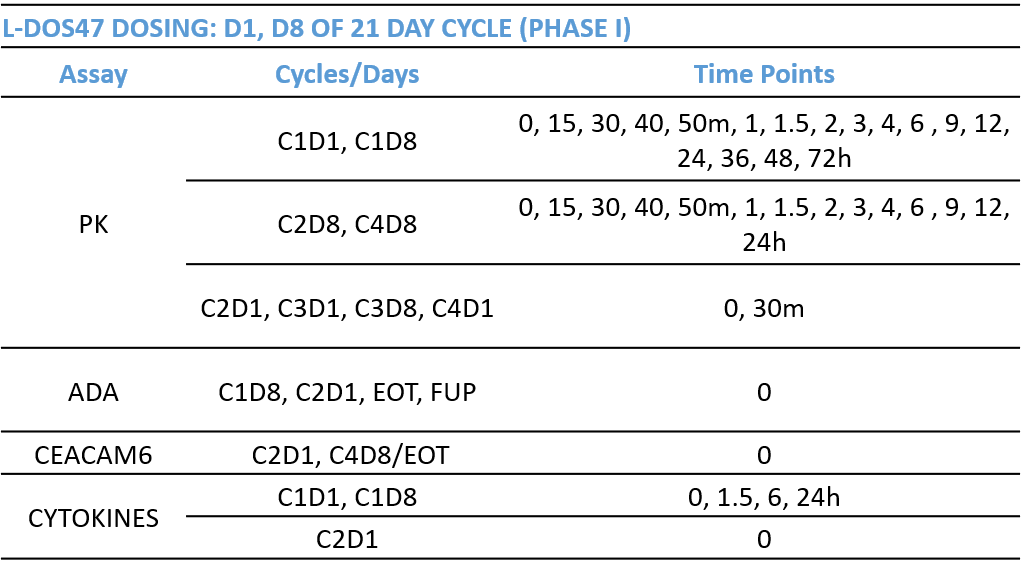


**Figure S1.** Study schematic for (*A*) Phase I and (*B*) Phase II.

H

F

G

E

D

B

C

A

**Figure S2.** Grouped by dose cohort, L-DOS47 mean C_max_ after (*A*) initial (C1D1), (*B*) C1D8, (*C*) C2D8 dosing, and (*D*) C4D8 dosing; L-DOS47 mean AUC_(0-t)_ after (*E*) initial (C1D1), (*F*) C1D8, (*G*) C2D8 dosing, and (*H*) C4D8 dosing.

**Figure S3.** Spearman correlation between half-life (h) and L-DOS47 dose (µg/kg) after initial (C1D1) dosing (r = 0.3117, two-tailed P = 0.1939).

A

B

**C D**

**Figure S4.** L-DOS47 immunogenicity. L-DOS47 AUC_(0-t)_ versus ADA titer over time in (*A*) all patients from the 0.21 – 13.55 µg/kg dose cohorts, and (*B*) Phase II patients dosed with 13.55 µg/kg twice weekly. (*C*) Cycle 1 Day 8 (C1D8) L-DOS47 ADA titers for Phase I Quartiles 1-4 versus Phase II (P2, n.s.: not significant; *p=0.0125; **p=0.0020). (*D*) C2D1 L-DOS47 ADA titers for Phase I Quartiles 1-4 versus Phase II (n.s.: not significant; *p=0.018; **p=0.0028).

**Figure S5.** Relationship between plasma CEACAM6 over time and BORR. Multiple Wilcoxon tests showed no significant changes from C2D1 to C4D8/End of Treatment (EOT).


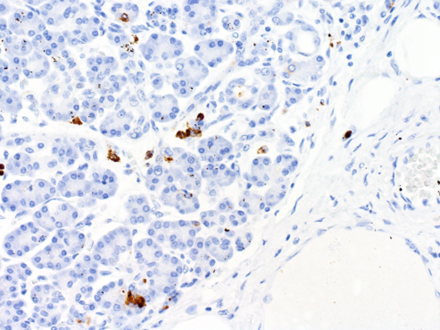

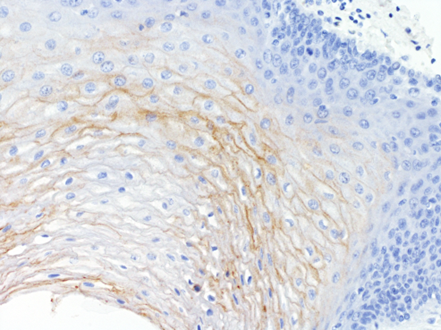

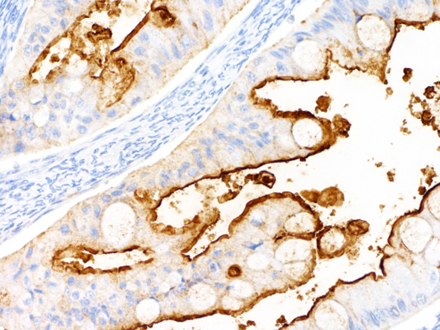

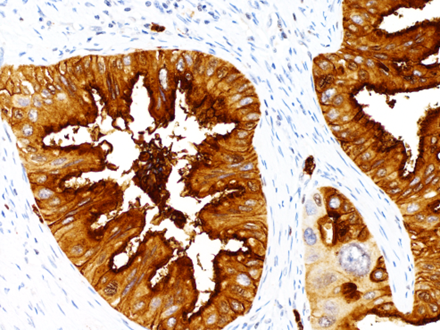


Score 0: negative in normal pancreatic epithelium,

scattered myeloid cells showing staining

Score 1: barely visible, weak membranous staining in the intermediate layer of normal squamous epithelium

Score 2: carcinoma with strong membranous staining without significant cytoplasmic staining

Score 3: carcinoma with strong membranous and

cytoplasmic staining

**Figure S6.** Scoring intensity examples for CEACAM6 immunohistochemistry staining. Examples are from (top to bottom): a normal tissue microarray, squamous tissue, pancreatic ductal adenocarcinoma tissue microarray (n=2).
